# Flow Injection-Based Refractive Index Sensing with a Si_3_N_4_ Photonic Crystal Nanobeam-Microring Fano Resonator

**DOI:** 10.1021/acsaom.5c00500

**Published:** 2025-12-09

**Authors:** Jesus Hernan Mendoza-Castro, Silvia Schobesberger, Artem S. Vorobev, Simone Iadanza, Giovanni Magno, Liam O’Faolain, Bernhard Lendl, Marco Grande

**Affiliations:** † Department of Electrical and Information Engineering, 18951 Politecnico di Bari, Via E. Orabona, 4, 70126 Bari, Italy; ‡ Institute of Chemical Technologies and Analytics, 27259 TU Wien, Getreidemarkt 9/164, Vienna 1060, Austria; § Institute of Applied Synthetic Chemistry, TU Wien, Getreidemarkt 9/163, Vienna 1060, Austria; ∥ Centre for Advanced Photonics and Process Analysis, 587895 Munster Technological University, Bishopstown, T12 T66T Cork, Ireland; ⊥ Tyndall National Institute, T12 PX46 Cork, Ireland; # Laboratory of Nano and Quantum Technologies, 28498 Paul Scherrer Institut, 5323 Villigen, Switzerland; ∇ Laboratory of Integrated Nanoscale Photonics and Optoelectronics, École Polytechnique Fédérale de Lausanne, 1015 Lausanne, Switzerland

**Keywords:** Fano resonances, flow injection analysis, refractive index sensing, microfluidics, silicon nitride

## Abstract

Asymmetric optical resonances shaped by Fano interference can provide enhanced signal contrast and tunable lineshapes in integrated photonics. However, realizing and exploiting these effects in fabricated devices remains challenging, as nanofabrication often reduces asymmetry and slope steepness. In this work, we experimentally demonstrate a hybrid silicon nitride photonic crystal nanobeam-microring resonator (PhCN-MRR) for refractive index (RI) sensing and systematically analyze how Fano-induced line shape asymmetry impacts transduction under realistic flow conditions. Despite exhibiting only moderate asymmetry, the PhCN-MRR shows distinct optical advantages for intensity-based detection, demonstrated using glucose solutions as a model analyte. The sensor was integrated into a custom Si_3_N_4_ microfluidic platform and tested under both stopped-flow and dynamic flow-injection conditions over concentrations from 0.5 to 10 mg/mL. Time-resolved spectral scans (0.5–1 Hz) enabled dual transduction channels via wavelength shifts (Δλ) and fixed-wavelength intensity changes (Δ*I*). Compared with a conventional microring resonator (MRR) featuring symmetric Lorentzian responses, the PhCN-MRR exhibited comparable Δλ sensitivities (∼111 to 113 nm/RIU) but delivered enhanced Δ*I* responsivity and improved contrast at low concentrations (e.g., 2 mg/mL) due to its asymmetric resonance slopes. These results link controlled Fano asymmetry to measurable sensing gains, demonstrating how modestly asymmetric resonances can improve real-time refractometric detection and expand the design space of Si_3_N_4_ photonic platforms for lab-on-chip analytical applications.

## Graphical abstract

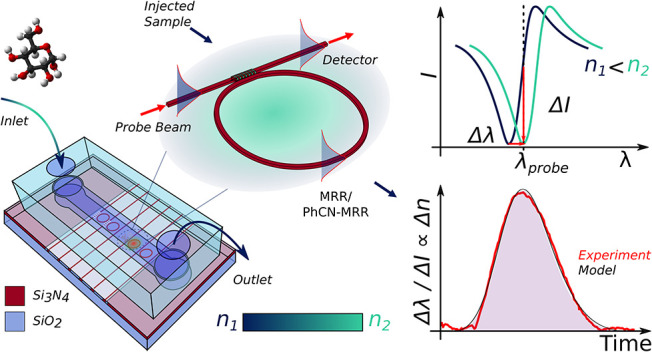

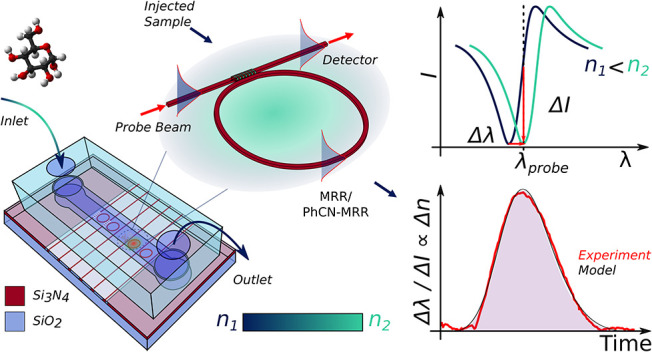

## Introduction

Fano resonances, arising from the interference between discrete resonant modes and a broadband continuum, are characterized by sharp, asymmetric lineshapes that offer unique advantages in integrated photonic systems.^1–3^ These interference-based resonances have been widely studied in integrated photonics, where their sharp spectral features have been exploited for functionalities such as optical switching,^4^ coherent light generation,^5^ directional signal routing,^6^ and sensitive detection of chemical and biological targets.^2^

The fundamental measurement principle governing all sensing schemes is the detection of refractive index (RI) changes in gas or liquid in proximity to or in contact with the resonant photonic structure, resulting in shifts in resonance frequency and/or resonance shape. Achieving chemical selectivity in complex matrices, such as liquids, often requires specialized surface functionalization.^7–11^ However, the essential transduction step remains the accurate measurement of the smallest changes in RI caused by the analyte. The sensitivity and precision of this measurement are directly determined by the optical response and line shape of the resonator, making them central to sensor design.

Among established RI-sensing platforms, microring resonators (MRRs) and photonic crystal nanobeam cavities (PhCNCs), offer high-quality factors (*Q*), narrow line widths, and small device footprints.^2,3,12–14^ Several hybrid architectures have emerged in recent years to induce asymmetric lineshapes, similar to Fano interference, by coupling resonators with photonic crystals, Mach–Zehnder interferometers, or nested cavities.^15–30^ Although these designs can enhance sensitivity or resolution, they often come at the expense of fabrication complexity, limited operational tunability, or reduced performance in aqueous environments, all of which are critical constraints for practical lab-on-a-chip biosensing implementations.^31,32^

Further, high-Q Fano resonances have also been demonstrated in free-space platforms such as metasurfaces and photonic crystal slabs.^33,34^ While these are highly effective in tailoring light-matter interactions from free space, our focus is on chip-scale devices. Such integrated architecture provides the critical advantages of seamless microfluidic integration, robust guided-wave operation without alignment, and scalable fabrication, all essential for practical (bio)-sensing applications.

In this work, we present a simplified Si_3_N_4_-based photonic sensor that integrates an MRR with a rectangular photonic crystal nanobeam (PhCN-MRR). This hybrid design introduces a controlled spectral perturbation in the bus waveguide, leading to a moderate but well-defined and reproducible asymmetry relative to a Lorentzian resonance. Although the degree of asymmetry is modest, the engineered line shape significantly alters the optical transduction behavior in both gaseous and aqueous matrices. Importantly, the device is fabricated in a single-etch step process without multilayer deposition or etch-stop techniques, offering scalability and compatibility with standard foundry-grade Si_3_N_4_ photonics.^35,36^

To assess the functional advantages of this resonance asymmetry, we implement a flow-injection analysis (FIA) setup to study dynamic RI transduction under realistic liquid-delivery conditions. By integrating a microfluidic channel directly on the chip, we enable reproducible injection of aqueous glucose solutions into the optical sensing region and capture the spectral evolution of the resonant structure over time. Beyond the traditional wavelength-shift (Δλ) metric, we extract fixed-wavelength intensity variations (Δ*I*) from time-resolved spectra, revealing enhanced responsivity during flow without active feedback or tracking.^37^

We investigate the role of engineered resonance asymmetry in enhancing sensitivity and transduction performance under realistic flow conditions. Thereby, we demonstrate a scalable, dual-regime sensing platform for real-time liquid-phase refractometric detection with improved interpretability and analytical flexibility. Owing to its robustness and the maturity of the Si_3_N_4_ photonics platform, the PhCN-MRR concept is relevant for compact liquid-phase analytical systems,^38^ including environmental monitoring and point-of-care assays where rapid bulk-RI readout is sufficient.

## Experimental Section

### Device Design and Fabrication

The sensor platform consists of two main configurations: a conventional microring resonator (MRR) and a modified structure in which a rectangular photonic crystal nanobeam (PhCN) is inserted within the bus waveguide, referred to as the PhCN-MRR (Figure 1a,b). Both devices share a nominal ring radius of *R*_MRR_ ≈ 16 μm, a bus-to-ring coupling length *L*_C_, and a vertical gap *g*_y_ = 0.35 μm. The distinguishing parameter in the PhCN-MRR is the periodic photonic crystal section length of the PhCN, defined by the number of rectangular slots *N*_H_ = 5 and their period *a* = 0.55 μm. Here, *L*_C_ is defined by *N*_H_ *a*.

**Figure 1:**
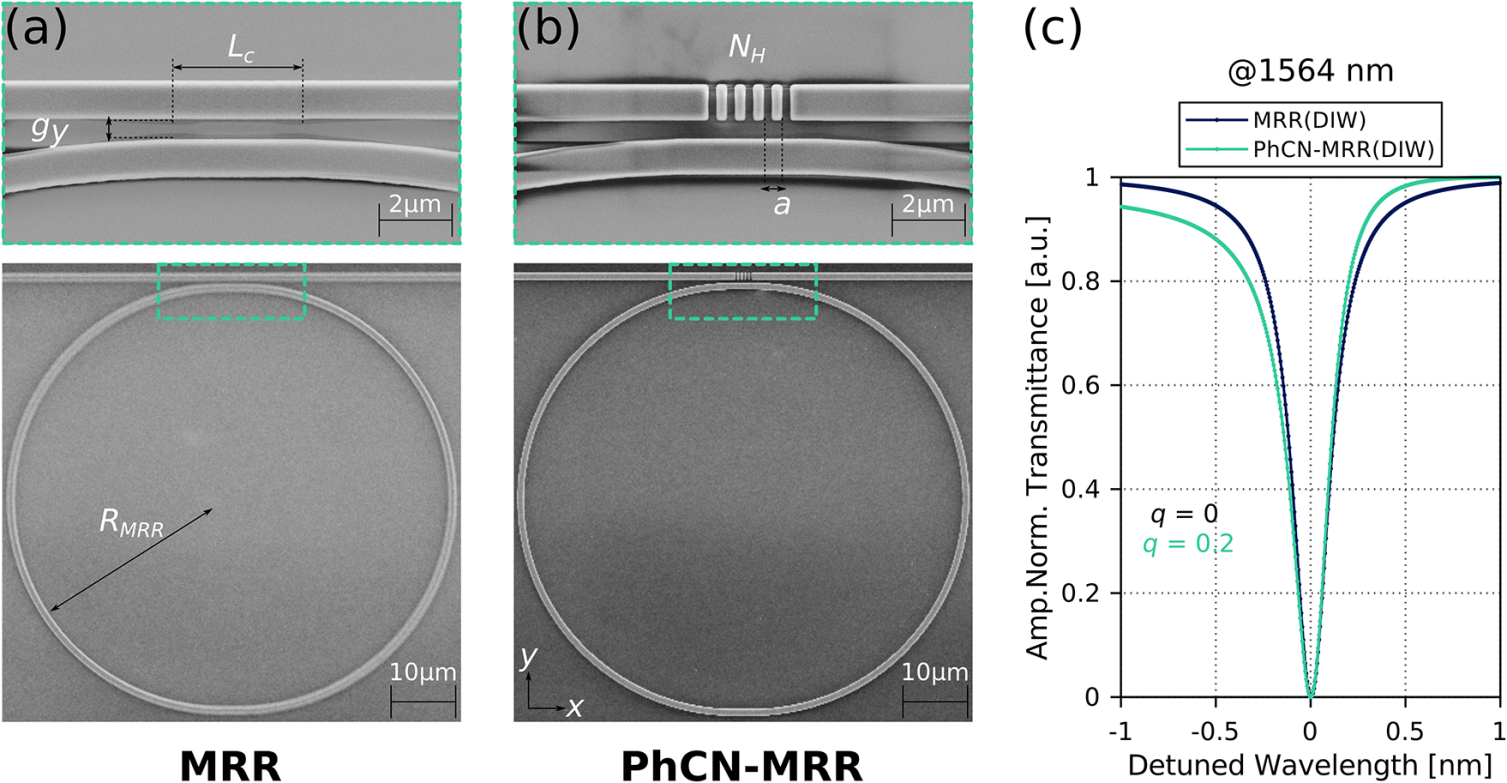
(a) SEM image of a fabricated Si_3_N_4_ microring resonator (MRR). (b) SEM image of a rectangular slot-based photonic crystal nanobeam-MRR (PhCN-MRR). Both devices share the same ring radius *R*_MRR_, vertical coupling gap *g*_y_ = 0.35 μm and coupling section length *L*_C_. The PhCN-MRR integrates a PhCN with a length defined by *N*_H_ = 5-unit cells and periodicity *a* = 0.55 μm. The inset shows a magnified view of the coupling region. (c) Fitted transmission spectra from experimental resonances of the two devices under DI water (DIW) cladding, centered around 1564 nm. The conventional MRR exhibits a symmetric Lorentzian profile with Fano parameter *q* = 0, while the PhCN-MRR displays a slightly asymmetric Fano line shape with *q* = 0.2.

These slots perturb the effective propagation constant of the bus waveguide and introduce a broadband nonresonant transmission pathway that interferes with the narrowband microring mode. The resulting interference produces a quasi-Fano line shape whose asymmetry is quantified by the dimensionless Fano parameter *q*. The photonic crystal parameters (e.g., *N*_H_ = 0–10) and the coupling strength (e.g., *g*_y_ = 0.25–0.55 μm) were varied to modulate the asymmetry while keeping the remaining design variables constant. All devices are designed to operate with the TE-like guided mode at 1550 nm under both gaseous and liquid matrices following similar design rules of our early work in.^13^

The asymmetric resonance lineshapes observed in our devices can be modeled using a modified Fano resonance function, commonly used in photonics to capture the interference between a discrete resonant state and a broad spectral continuum:^18,39,40^

1 $$F\left(\omega \right)=A_{0}+F_{0}\frac{{\left[q+2\left(\omega -\omega_{0}\right)/\Gamma \right]}^{2}}{{1+\left[2\left(\omega -\omega_{0}\right)/\Gamma \right]}^{2}}$$

here ω is the incident optical frequency, ω_0_ is the center resonant frequency, and Γ represents the resonance line width. The parameter *F*_0_ is a scaling factor, *A*_0_ denotes a vertical offset, and *q* is the unitless Fano asymmetry parameter that characterizes the degree of spectral skewness. When *q* = 0, the resonance profile becomes a symmetric dip (Lorentzian), while increasing or decreasing *q* leads to asymmetric lineshapes.

Figure 1c shows representative measured spectra for a MRR and a PhCN-MRR structure. The MRR spectrum is well described by a Lorentzian fit (*q* ∼ 0), whereas the PhCN-MRR spectrum exhibits a moderate asymmetry consistent with a fitted value of *q* ∼ 0.2, confirming the perturbative influence of the PhCN section on the resonance shape.

The total quality factor *Q* for each resonance is extracted from the fitted parameters as *Q* = ω_0_/Γ. These values, along with the extinction ratio (ER) and asymmetry (*q*), are used to compare obtained results when using the PIC devices under different experimental conditions. The ER is defined as ER­(dB) = 10·log_10_(*T*_max_/*T*_min_), with *T*_max_ and *T*_min_ being the maximum and minimum normalized transmittance values of the resonance curve, corresponding to the off-resonance (baseline) and on-resonance (dip) transmission levels, respectively.

Devices were fabricated on 4″ silicon wafers thermally oxidized and coated with 300 nm of Si_3_N_4_ deposited by plasma-enhanced chemical vapor deposition (PECVD). A ∼ 450 nm-thick ZEP 520A electron-beam resist was spin-coated and patterned via 100 kV electron beam lithography (EBL). After development in n-Amyl Acetate and rinsing with IPA, patterns were transferred into the Si_3_N_4_ layer using inductively coupled plasma (ICP) etching with a O_2_:CHF_3_ chemistry in an 8:42 ratio. Devices were fabricated using high-resolution EBL followed by an optimized ICP-RIE process providing high etch selectivity and near-vertical sidewalls (∼90°). Sidewall roughness and etch quality were verified through SEM inspection.

Resist removal was performed via O_2_ plasma ashing followed by immersion in MICROPOSIT 1165. The wafers then underwent sequential cleaning in acetone, and IPA to complete the fabrication process. All devices were realized in a single full-etch step, ensuring compatibility with scalable, foundry-grade photonics processes.

The choice of Si_3_N_4_ is driven by its low thermo-optic coefficient which ensures spectral stability (e.g., ambient temperature fluctuations), and reduced RI contrast with cladding (e.g., air or SiO_2_) that enables low propagation losses, critical for achieving high-quality factor resonators. Furthermore, its negligible two-photon absorption at telecom wavelengths prevents nonlinear effects, a common issue in silicon-based platforms.

### Microfluidics Integration

Microfluidic sealing is a critical aspect of lab-on-chip platforms, particularly for photonic sensing applications where liquid delivery must remain stable and reproducible. In this work, a rapid, robust, and biocompatible microfluidic integration strategy was implemented, relying on a hybrid stack combining pressure-sensitive adhesive (PSA) tape and a PDMS layer.^41,42^

Figure 2a–c illustrates the stack layout employed for integration. A transparent double-sided PSA tape (MA-93 acrylic adhesive with a 12.7 μm PET core and total thickness of 48.26 μm including liners) was used to define the microfluidic channel (Figure 2a). The channel geometry was cut using a vinyl cutter, allowing the free definition of the channel opening. A square PDMS layer, die-cut to match the chip dimensions, was prepared with inlet and outlet holes (diameter ∼ 1.5 mm). After plasma activation of both PSA and PDMS, the two layers are bonded (Figure 2b), and the assembly was aligned and laminated on top of the Si_3_N_4_ photonic chip (Figure 2c).

**Figure 2:**
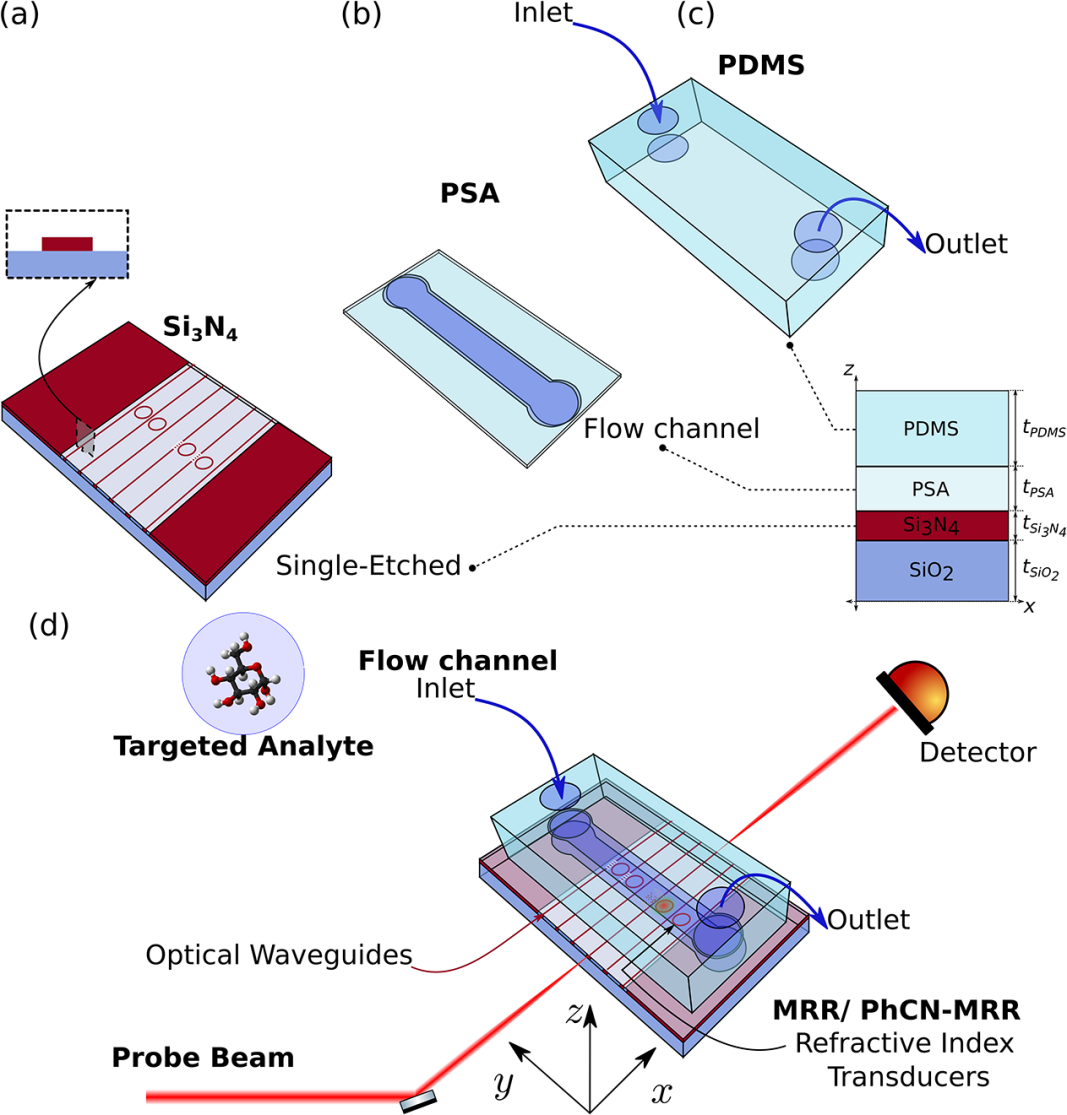
(a) A Si_3_N_4_ chip with single-etch nanophotonic structures. (b) Microstructured pressure-sensitive adhesive (PSA) layer, defining the microchannel architecture. (c) Cut polydimethylsiloxane (PDMS) foil with mechanically punched inlet/outlet ports. Right: schematic cross-section showing the stacked integration (PDMS/PSA/Si_3_N_4_/SiO_2_), where *t*_PDMS_, *t*_PSA_, *t*_Si_3_N_4__, *t*_SiO_2__ indicate the corresponding thicknesses. (d) 3D schematic of the final bonded assembly, where the PSA+PDMS microfluidic module delivers the analyte solution to the RI transducers (MRR and PhCN-MRR). Simultaneously, a probe laser beam is edge-coupled into the chip waveguides for continuous optical interrogation during flow injection analysis.

Unlike conventional microfluidic bonding techniques that rely on localized oxide removal or multilayer deposition, this approach simplifies the assembly and reduces fabrication time to under few hours. If further strength of the bonding is required, ∼12 h clamping is sufficient. Importantly, this method avoids the need for deposited SiO_2_ cladding and its subsequent patterning. The use of PSA ensures uniform adhesion, reduces delamination risks, and is compatible with high-throughput testing. Leak tests with a peristaltic pump at 132 μL/min for 5.5 h confirmed the mechanical stability of the assembled flow cell. No leakage was observed even after one month of storage, supporting the reliability of this approach.

The microfluidic layout is optimized to maintain a low dead volume and ensure fast, laminar delivery of liquid samples. The straight channel (10 mm long, 0.75 mm wide, 50 μm deep) aligns with the sensing region containing the set of MRR and PhCN-MRR cavities. The bonding strategy and vertical stack design allow the channel to run directly above the exposed resonator, ensuring high interaction between the guided optical mode and the surrounding refractive index, as demonstrated in Figure 2d. A probe beam is butt-coupled into the Si_3_N_4_ photonic integrated circuit, where light propagates through waveguide networks before evanescently side-coupling to the designed photonic cavities. The cavity claddings are selectively exposed to a microfluidic channel, enabling direct interaction with the sample injected into the flow system. At the output, a detector monitors changes in the intensity reflecting changes in the optical response of the resonators, transducing refractive index variations in the fluid into measurable signals.

This configuration is particularly suited for flow injection analysis (FIA), where the sample dispenses in the flowing carrier in a highly reproducible way, giving rise to typical FIA peaks. The precise control over the flow conduit geometry and flow rate are critical to ensure reading of transient RI changes of the sample in contact with the PIC transducer.

### Optical Characterization

The optical performance of the integrated sensor platform was evaluated first under stopped-flow conditions by comparing the transmission spectra of the devices when the microchannel was empty (air-clad) and filled with DIW (liquid-clad). This characterization was performed using the microfluidic integration stack described above. Spectral interrogation was carried out via end-fire coupling setup and a tunable laser source, with TE-like mode purity enhanced through polarization control. Supporting Information, Section A, provides further details on the implemented setup.

#### Static Resonance Response and Key Metrics

Figure 3a,b shows representative spectral responses for the two key configurations: a MRR (*N*_H_ = 0) and a PhCN-MRR (*N*_H_ = 5), both with a fixed coupling gap of *g*_y_ = 0.35 μm and *L*_c_ = 2.75 μm. Here, insertion loss refers to the total optical power loss through the device under stopped-flow, steady-state conditions, and is used to evaluate baseline transmission and resonance contrast before dynamic sensing measurements. In Figure 3a, the MRR shows a consistent series of Lorentzian resonances across the S+C+L Telecom bands. As expected, the spectral response under DIW (teal trace) exhibits a red shift compared to air (black trace), due to the higher cladding refractive index. Additionally, the ER improves at longer wavelengths (>1520 nm), approaching ∼ 30 dB near critical coupling.

**Figure 3:**
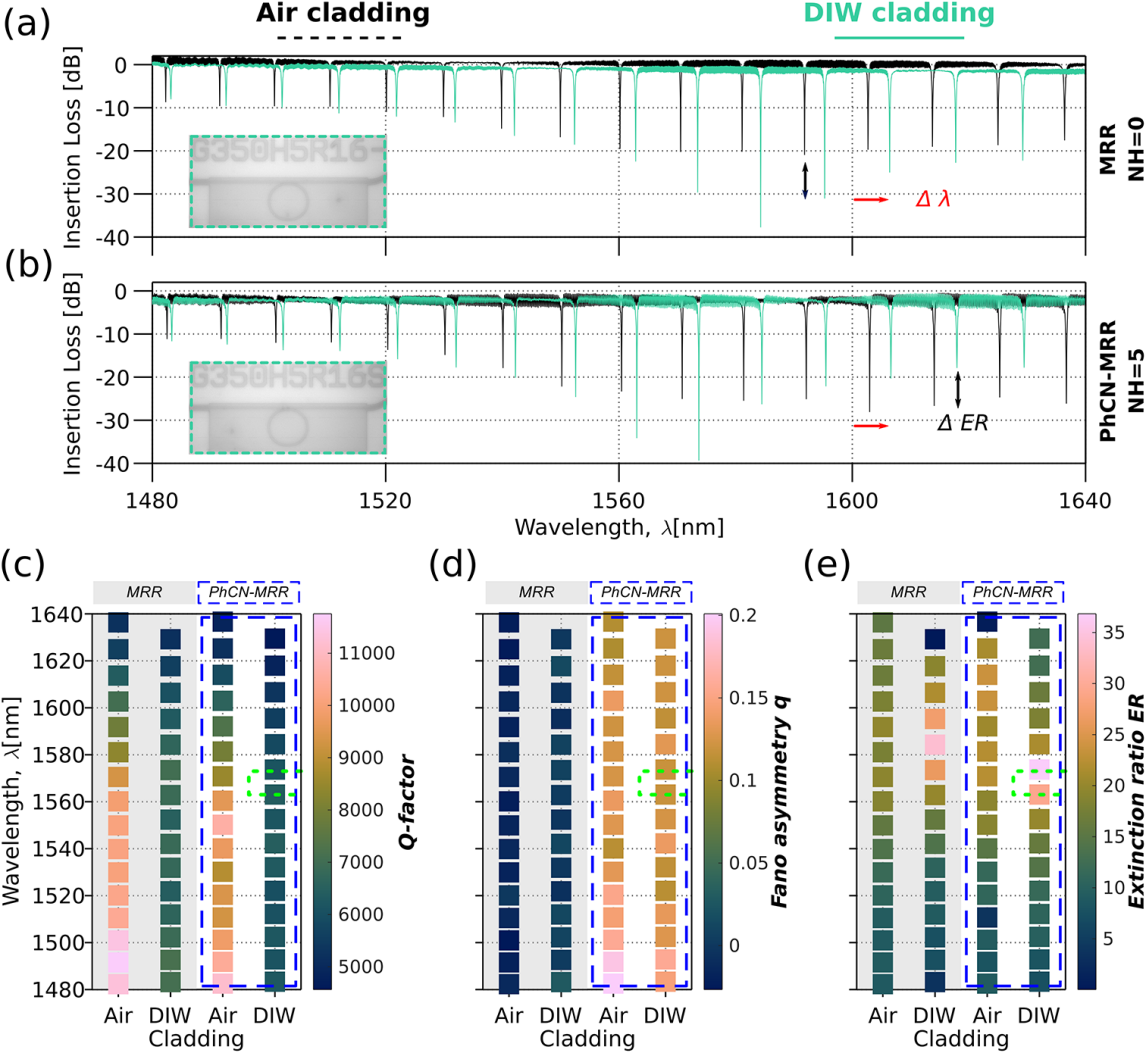
Spectral response and resonance characterization of the hybrid PhCN-MRR versus the reference MRR. (a, b) Measured transmittance of a PhCN-MRR with *N*_H_ = 5, under (a) air cladding and (b) deionized water (DIW) cladding. All devices share a fixed coupling gap *g*_y_ = 0.35 μm and coupling length *L*_C_ = 2.75 μm. (c–e) Heat maps of the extracted resonance metrics; (c) Q-factor (*Q*), (d) Fano asymmetry parameter (*q*), and (e) extinction ratio (*ER*); for all resonances across the measured wavelength range. Each panel displays side-by-side results for the reference MRR and the PhCN-MRR under two cladding conditions (Air and DIW), with column groups explicitly labeled. The gray-shaded columns denote reference MRR values, while the dashed blue boxes mark the PhCN-MRR spectra. The green dashed circles highlight the specific resonance evaluated in the main experiments.

In Figure 3b, the PhCN-MRR (*N*_H_ = 5) exhibits more asymmetric resonances and a broader spectral extinction window. Again, the DIW-clad configuration shows a red-shift and enhanced ER, with the strongest dips occurring between 1560–1585 nm. The lower transmission baseline in the PhCN-MRR compared to the MRR reflects added scattering and dissipation introduced by the periodic slots.

Notably, in both devices, the DIW-clad configuration reveals a distinct region of peak extinction, indicative of quasi-critical coupling,^43^ followed by a reduction in ER at longer wavelengths. This trend is more pronounced in the PhCN-MRR, confirming the design-induced sensitivity to cladding perturbations. A top-view microscope image of the channel filled with DIW is shown inset in Figure 3a,b, confirming uniform fluidic filling during interrogation.

To assess resonance quality, we extracted three key parameters from the Fano fitting: the quality factor, extinction ratio, and Fano asymmetry parameter. While the *Q*-factor is widely used in the photonics community as a standard performance metric, in this work we emphasize the role of intensity contrast, which directly impacts the signal-to-noise ratio in sensing.^3^ This aligns with previous studies suggesting that the product of *Q*-factor and intensity contrast *I* (*Q* × *I*) serves as a useful figure-of-merit (FOM) for sensing.^3,12^ Here, intensity contrast is express as ER (dB), which captures the resonance depth in the logarithmic scale. For a fixed resonance *Q*, an increased ER leads to stronger intensity contrast at the resonance slope, improving the signal-to-noise ratio in intensity-based sensing. While ER is expressed here in dB, its logarithmic nature still preserves the relative ranking of transduction performance. Therefore, within constant-*Q* designs, a higher ER corresponds to improved sensing FOM. We note that resonances near 1564 nm exhibit both high *Q* and large ER (>25 dB), making them particularly attractive for sensitive RI detection.

#### Resonance Metrics Mapping

Figure 3c–e summarizes the extracted resonance parameters, *Q*-factor, ER, and Fano asymmetry *q*, for both the reference MRR and the hybrid PhCN-MRR under air and DIW cladding.

To analyze resonance characteristics (e.g., *Q*, *q*, ER) across devices, we represent extracted spectral data as heat maps. Each data point corresponds to a single resonance from a specific device (e.g., MRR), indexed by cladding material (Air or DIW) and wavelength (1480–1640 nm). The vertical axis denotes λ, while the horizontal axis categorizes devices into reference MRRs and slot-engineered PhCN-MRRs. Color intensity encodes the magnitude of each metric (e.g., *Q*), with higher values (e.g., quality factor) indicated by a brighter color, as defined by the adjacent color bar. Dashed bounding boxes group devices by type, facilitating intra- and intergroup comparisons of cladding- and wavelength-dependent performance variations.

Under air cladding, both devices exhibit higher *Q*-factors compared to their DIW counterparts, with values exceeding 10^4^ at several resonances. A general decreasing trend of *Q* with wavelength is observed across both configurations (Figure 3c), consistent with dispersion effects and increased absorption at longer wavelengths.

As expected, the *q* parameter remains close to zero for the standard MRR across all wavelengths, consistent with its Lorentzian line shape. In contrast, the PhCN-MRR shows significantly higher *q* values, confirming the emergence of Fano-like asymmetry due to the PhCN insertion ( Figure 3d). This is particularly evident in Figure 3b, where resonances above 1573 nm are suppressed (lower ER) compared to the air-clad case, while those at shorter wavelengths show both higher ER (>25 dB) and moderate asymmetry (*q* ∼ 0.2).

Although the *Q*-factor is slightly reduced in the DIW-clad PhCN-MRR, the ER improves notably for resonances below 1570 nm, often surpassing 10 dB. This trade-off reflects a key design principle in this platform: prioritizing strong intensity contrast, even at the expense of modest *Q* reduction. This motivates the selection of resonances with both high ER and moderate *q* values (e.g., near 1564 nm), where the line shape provides both strong signal contrast and sufficient slope for tracking RI variations.

Figure 3e presents the corresponding heat map of the extinction ratio, visually confirming the clustering of high-ER resonances in the sub-1570 nm range for the DIW-clad PhCN-MRR. These metrics confirm that the PhCN-MRR sustains robust, asymmetric resonances in liquid environments while offering improved extinction ratios, a key requirement for high-contrast optical sensing.

The observed high Q-factor, coupled with an anticipated moderate sensitivity, stems from the strong field confinement within the Si_3_N_4_ core. This localization minimizes scattering loss but reduces the evanescent field overlapping with the analyte, a characteristic trade-off in this platform. Simulated field distributions of the device are provided in Supporting Information, Section D.

The same principle of modal confinement also explains the dynamic performance tuning achieved via the cladding (See Figure 3 for cladding RI change). The enhanced ER in the DIW scenario can likely be attributed to the increased RI of the cladding. This further improves modal confinement in both the MRR and the PhCN, which sharpens the contrast between the two interfering pathways and consequently increases the ER.

### Flow Injection Analysis Methodology

Following the optical characterization of the proposed device under aqueous cladding and stopped-flow conditions using the integrated microfluidics setup, the RI sensor suitability for recording dynamic refractive index changes was further investigated using a flow injection analysis approach.

Flow injection analysis (FIA) is a well-established and versatile technique in chemical sensing, based on the injection of a sample into a continuously flowing carrier stream that transports it to a detector operated in flow-through mode. During transport, the sample undergoes a well-defined dispersion caused by radial and axial diffusion under laminar flow conditions. As the diluted sample plug reaches the detector, it generates a highly reproducible flow injection peak. This dynamic profile enables the sensor to interact with a continuous range of concentrations as the sample passes through. Since the baseline signal can be monitored both before and after the peak, drift or offsets in the measurement can be identified and corrected. Owing to its simplicity, control, and repeatability, FIA is particularly well suited for sensor characterization, allowing automated and reproducible analyte-sensor interaction under defined conditions.

The FIA manifold consisted of a peristaltic pump fitted with Teflon pump tubing, PTFE flow channels with an inner diameter of 0.5 mm, and a six-port injection valve (Figure 4a). For consistent and reliable comparison between sensor configurations, a standardized FIA setup was implemented. A fixed 100 μL volume of glucose solution was injected into a continuous background flow (carrier) of deionized water (DIW) at a flow rate of 70 μL/min through the microfluidic channel enclosing the sensor chip. Time-resolved spectral monitoring was performed using a tunable semiconductor laser (TSL) under continuous flow conditions. The detailed workflow of the injection protocol and liquid handling configuration is presented in Supporting Section A.

**Figure 4:**
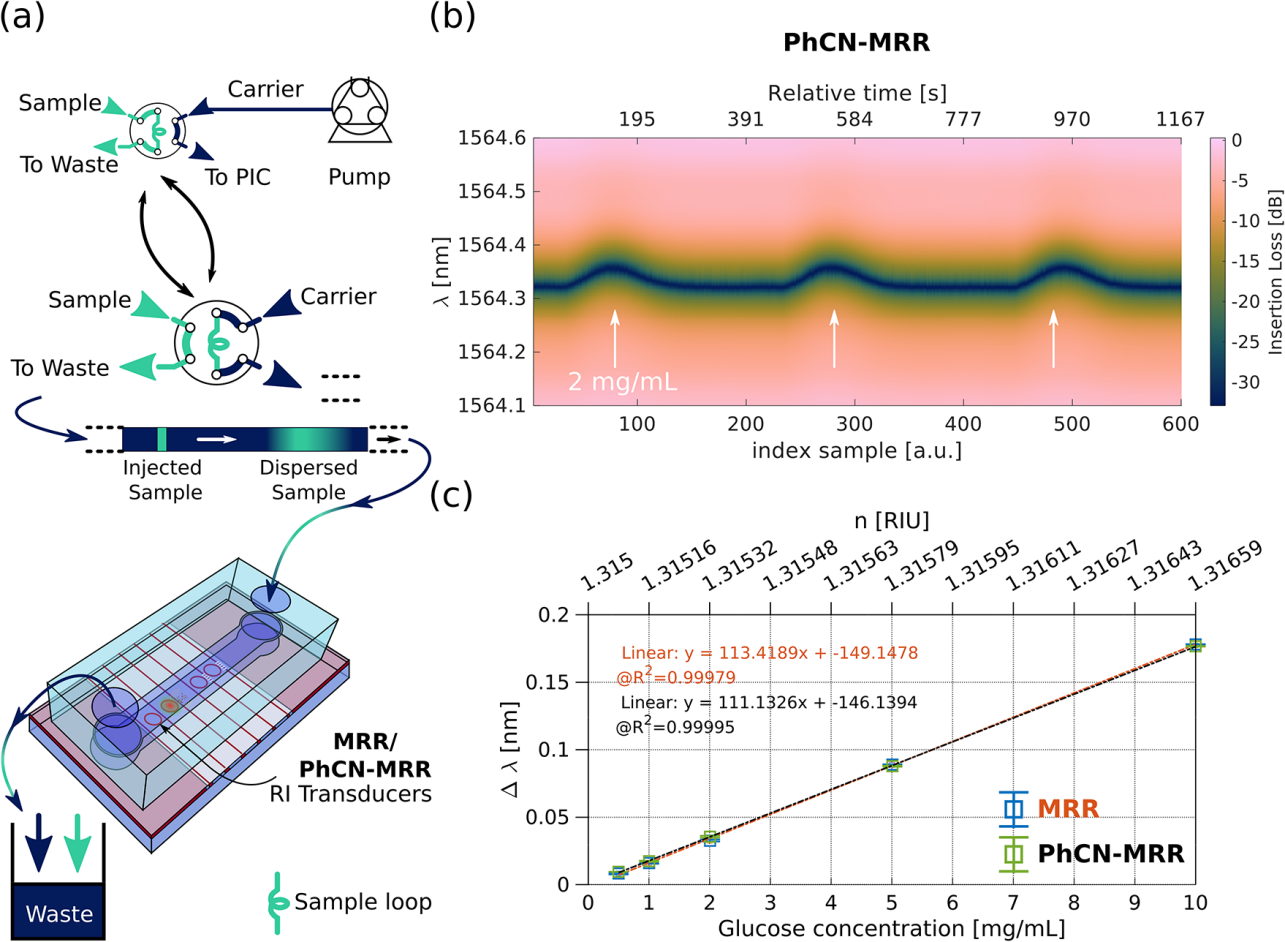
Flow injection analysis (FIA) using integrated MRR and PhCN-MRR devices for refractive index sensing. (a) Schematic of the FIA-based liquid handling system used for real-time refractive index sensing. The setup includes a six-port injection valve for controlled sample loading, a peristaltic pump for carrier flow, and PTFE tubing connecting to a microfluidic channel enclosing the PIC sensor. The system operates in continuous-flow, allowing stable sample dispersion under laminar flow for concentration gradient generation. (b) Time-resolved transmission spectra for the PhCN-MRR under a 100 μL injection of 2 mg/mL glucose at 70 μL/min, showing three eluted FIA peaks. (a) Measured sensitivity for a conventional MRR (non-Fano) and PhCN-MRR (Fano) with *g*_y_ = 0.35 μm, *N*_H_ = [0; 5] and *L*_C_ = 2.75 μm using glucose solutions (0.5–10 mg/mL). Each concentration was tested in at least three consecutives runs (typically five), and the average shift was used to generate the calibration curves. Corresponding error bars in (c) reflect the standard deviation across repeated measurements, accounting for minor fluctuations in injection and measurement precision.

Upon injection, the sample experienced hydrodynamic dispersion due to laminar flow and Taylor-Aris diffusion, resulting in a broadened sample zone. This led to a characteristic FIA peak with a dilution factor *D* = *c*/*c*_0_ = 0.7 at peak maximum, where *c*_0_ is the initial sample concentration and *c* is the effective concentration at the sensor during peak maximum.

Glucose was selected as the test analyte because the RI of aqueous glucose solutions as a function of concentration is well understood and exhibits stable, monotonic behavior, making it a standard benchmark for bulk RI sensor calibration. This ensures that the sensor response originates purely from RI modulation rather than surface binding.

## Results and Discussion

### FIA Protocol and Sensing Workflow

These FIA experiments were conducted to compare the sensitivity and signal response of the PhCN-MRR (Fano) and conventional MRR (non-Fano) designs to small RI variations, using glucose as a test analyte.

During each injection, resonance spectra were continuously recorded by sweeping the TSL over a 1 nm range centered on the selected resonance. This enabled time-resolved spectral acquisition at a rate of approximately 1 Hz. The recorded spectrum was then postprocessed to extract the dynamic response associated with each FIA peak.

The experiment leveraged both wavelength shift (Δλ) and intensity change (Δ*I*) to evaluate performance for these two detection strategies. Tracking Δλ provides insights into waveguide sensitivity^13,23^ while monitoring the Δ*I* at the resonance IP captures slope driven contrast, directly linked to the steepness and asymmetry discussed previously.

Figure 4b,c summarizes key experimental results. As an illustrative case, Figure 4b shows the time-resolved transmission profile for the PhCN-MRR during a 2 mg/mL glucose injection, representative of the broader concentration series. The spectral sensitivity of the MRR and the PhCN-MRR was evaluated over five glucose concentrations (0.5–10 mg/mL), from the spectra recorded at the FIA peak maxima. Measurements were performed at resonances of 1585 and 1564 nm, respectively. As shown in Figure 4c, measured sensitivities were ∼113 nm/RIU for the MRR and ∼111 nm/RIU for the PhCN-MRR. The variation between the two was less than 2%, indicating nearly identical Δλ response. This suggests that introducing the PhCN, and the resulting Fano effect, does not significantly alter the bulk sensitivity of the MRR. This is consistent with the fact that the effective RI of the guided mode in the MRR waveguide continues to dominate the wavelength shift.

### Transduction Metrics

From the time-resolved resonance profiles, two key transduction metrics can be directly extracted: the wavelength shift (Δλ) and the intensity change (Δ*I*). Figure 5a shows the spectral evolution during a representative 2 mg/mL glucose FIA peak, with vertical lines tracking the time-dependent position of each resonance. The resonance minima, marked with circles, define the Δλ trajectory, which is plotted in Figure 5b for both the PhCN-MRR (black filled) and the MRR (orange dashed).

**Figure 5:**
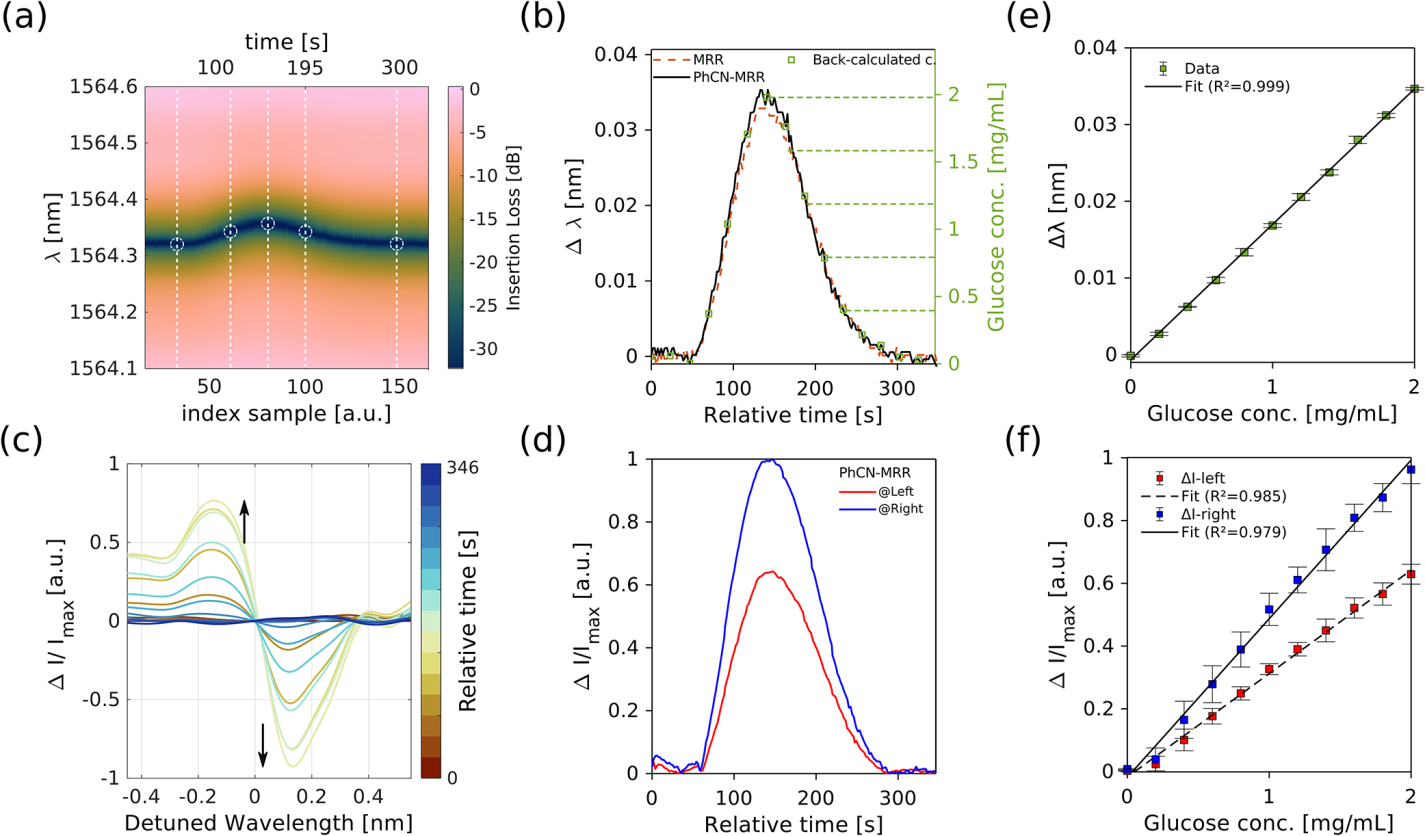
Transduction signal extraction from a 2 mg/mL glucose injection (FIA peak). (a) Zoomed-in spectral evolution during the first FIA peak, with vertical lines indicating the time evolution of individual resonances. Circles mark the extracted minima (resonance wavelengths), used to compute the wavelength shift Δλ. (b) Extracted Δλ traces for both devices. The primary vertical axis shows the wavelength shift, while the secondary axis displays the back-calculated glucose concentration derived from the calibration curve in Figure 4. (c) Amplitude differences of the normalized transmission spectra during the FIA peak, computed by subtracting the baseline spectrum at the start of the injection. (d) Extracted Δ*I* values for the left (red) and right (blue) inflection points of the resonance dips. (e) Calibration curve for Δλ based on 11 concentration points (0–2 mg/mL) derived from the front tail of FIA peaks. (f) Corresponding Δ*I* calibration curves using the same concentration points, separately evaluated for left and right inflection points.

While both devices respond consistently to the glucose injection, the MRR exhibits slightly lower responsivity at this concentration. The secondary vertical axis in Figure 5b displays the back-calculated analyte concentration using the calibration curve from Figure 4c. Horizontal dashed lines illustrate concentration values inferred from the trailing edge of the FIA response, highlighting how plug dispersion during injection creates a local, time-dependent concentration gradient within each cycle.

Complementary to this analysis, we investigated the potential of intensity changes as an alternative transduction signal focusing on the PhCN-MRR. Figure 5c presents the differential intensity spectra obtained by subtracting the baseline (*t* = 0) spectrum from subsequent traces, revealing two distinct lobes on either side of the resonance. These features are most prominent in the PhCN-MRR due to its asymmetric Fano line shape. The left (negative slope) and right (positive slope) inflection points of the resonance correspond to the locations of maximum intensity change and were used to extract Δ*I*, as shown in Figure 5d.

Using these extracted signals, calibration curves were constructed based on 11 reference points derived from the front edge of FIA peaks for glucose concentrations ranging from 0 to 2 mg/mL. Figure 5e shows the Δλ-based calibration curve, and Figure 5f shows the corresponding Δ*I*-based curves for both left and right slopes. While Δλ offers excellent linearity and minimal drift in this setup, Δ*I* can provide enhanced responsivity but is more susceptible to alignment and power fluctuations.

A detailed comparison of the analytical performance for each method is presented in Table 1, including metrics such as slope, intercept, goodness of fit (*R* ^2^), Standard deviation of residuals (SD), Coefficient of variation (CV), limit of detection (LOD), and limit of quantification (LOQ). The Δλ method stands for the highest linearity *R* ^2^ and the lowest SD and CV. Note that the linearity for all methods is as high as 0.98. In contrast, Δ*I* stands for a reduced LOD and LOQ (3.5-fold) and an enhanced detection capability (28-fold) compared to Δλ.

**Table 1:** Analytical Performance Metrics for Wavelength (Δλ) and Intensity (Δ*I*) Based Transduction under FIA Conditions

transduction method	slope [signal/conc.]	intercept [signal]	*R* ^2^	^a^SD [signal]	^b^CV [%]	^c^LOD [mg/mL]	^d^LOQ [mg/mL]
Δλ [nm]	0.0177 nm/(mg/mL)	6.7731 × 10^–4^ nm	**0.9990**	**3.6342** **× 10^–4^** nm	**2.1392**	0.0832	0.2774
Δ*I*-left [a.u.]	0.3297 au/(mg/mL)	0.0174 au	0.9846	0.0265 au	8.4891	0.0485	0.1618
Δ*I*-right [a.u.]	**0.5055** a.u./(mg/mL)	0.0184 au	0.9794	0.0471 au	9.6669	**0.0238**	**0.0794**

a Standard deviation of residuals

b Coefficient of variation.

c Limit of detection (3σ).

d Limit of quantification (10σ).

These results confirm that both Δλ and Δ*I* provide viable channels for quantitative sensing under dynamic flow, with the PhCN-MRR showing advantages in intensity-based detection due to its sharper, asymmetric spectral profile. The availability of both wavelength- and intensity-based transduction offers flexibility in sensing strategies and the potential to optimize responsivity based on application-specific constraints.

While static and quasi-static measurements form a solid foundation for calibration and transduction metric extraction, real-time sensing introduces additional complexity related to signal fidelity, peak shape distortion, and temporal alignment. These dynamic behaviors are examined in the following sections, where FIA peak profiles are modeled and compared using metric-based fitting approaches

### Noise Sources Affecting Measurement Uncertainty and Error Mitigation

To complement the reported standard deviations, the dominant sources of uncertainty in the RI measurements and the corresponding mitigation strategies are summarized below:

#### Tunable Laser Source Resolution

Fluctuations in the probe source and the finite wavelength resolution of the tunable laser introduce uncertainty in the extracted resonance wavelength Δλ. The measured spectral noise floor ∼1 pm (set by the wavelength step size), and the TLS wavelength repeatability is ∼ ± 5 pm (typical). To minimize drift, the TLS was allowed to warm-up for 30–60 min prior to measurements.

#### Temperature Fluctuations

As Si_3_N_4_ and DIW have thermo-optic coefficients of opposite sign, even subdegree temperature variations can induce measurable resonance drifts.^44^ For typical Si_3_N_4_-DIW geometries, the resulting effective thermo-optic shift is on the order of tens of picometers per °C, depending on modal confinement. Experiments were performed in a temperature-controlled environment (±0.02 °C), and the microfluidic chip was allowed to reach thermal equilibrium for at least 30 min before measurements. The setup was enclosed to reduce environmental perturbations from ambient laboratory. Residual slow drift (time scale: tens of minutes) was removed using baseline referencing during the stop-flow intervals.

#### Intensity and Coupling Fluctuations

Source-power drift and fiber-to-chip coupling affect the Δ*I* metric. The TLS exhibited a power stability of ± 0.01 dB over 1 h, coupling remained stable during each ∼40 min measurement set. Coupling was reoptimized before each concentration series, and relative intensity variations were normalized to the off-resonance baseline.

#### Features Extraction Uncertainty

Δλ and Δ*I* were extracted directly from the raw spectra using subresolution interpolation methods, since model-based fitting was not sufficiently robust for the full data set. This avoids fitting artifacts but introduces uncertainty associated with spectral sampling, noise, and interpolation stability. Although no dedicated validation study was performed, repeated FIA injections produced consistent spectral minima and Δ*I* values, indicating that the extraction procedure was stable within the resolution limits of the system

#### Device-to-Device Variability

Sidewall roughness and small fabrication deviations may shift absolute resonance wavelengths; however, the refractive-index response of the Fano resonance is expected to remain qualitatively consistent across nominally identical devices. To avoid introducing interdevice variability into the analysis, all reported measurements were performed on the same device. Additional devices referenced in the manuscript were fabricated on the same chip and within the same lithography run.

### Reconstructed FIA Peaks

To establish the temporal characteristics of the sensing response, we first analyzed the reconstructed FIA signal profiles from both devices (PhCN-MRR and MRR) under dynamic flow. Representative traces of resonance Δλ during repeated glucose injections at selected concentrations (1–2 mg/mL) are presented in Supporting Section B, Figure S2, aligned to the known injection windows (shaded regions). Each FIA peak corresponds to a transient increase in analyte concentration caused by the injection of a 100 μL glucose sample into a continuous DIW background flow at 70 μL/min.

Both the conventional MRR and PhCN-MRR exhibit reproducible and well-defined FIA peaks with minimal baseline drift. Peaks consistently begin within 15–20 s of valve actuation and return to baseline after ∼ 140 s, reflecting the plug has full passage through the sensing region. While the general shape is preserved across replicates, slight asymmetries in FIA peak broadening are observed, consistent with dispersion at the leading/trailing edges during laminar flow.

Minor temporal offsets between replicates and between the two devices are attributed to manual valve actuation and the sequential (nonsimultaneous) interrogation setup. Crucially, signal baselines remain stable between injections, establishing a reliable reference for subsequent dynamic metric extraction.

These aligned and reproducible signals form the basis for the parametric modeling and metric-based comparison presented in next section, where Δλ and Δ*I* responses are quantitatively assessed via skew-normal peak fitting.

### FIA Peak Modeling and Metric-Based Comparison

In addition to direct time-domain analysis of Δλ and Δ*I* signals, we modeled each FIA peak using a skew-normal distribution to quantitatively capture their asymmetric shapes. Unlike symmetric Gaussian fits, which often fail to reflect the dispersion-induced tailing common in flow injection systems, the skew-normal approach offers a more flexible and physically relevant description.^45^

#### Skew-Gaussian Fitting of FIA Peaks

FIA peaks acquired under flow conditions inherently exhibit asymmetric profiles, typically with a sharp rising edge and slower decay. This behavior arises from the combined effects of convective transport, analyte diffusion, and channel dispersion.^45^ A skew-normal distribution is particularly well-suited to capture these features, offering three intuitive parameters, amplitude (area), width, and skewness, that respectively reflect signal strength, temporal spread, and asymmetry. Modeling the signal traces with this distribution enables robust quantification of each injection response and allows direct comparison across devices, concentrations and transduction magnitudes.

Each FIA peak was modeled using a skew-normal function of the form

2 $$S_{i}\left(t\right)=\frac{A}{\sqrt{2\pi \sigma_{i}^{2}}}\exp\left[-\frac{{\left(t-\tau_{i}\right)}^{2}}{2\sigma_{i}^{2}}\right]\left[1+\mathrm{erf}\left(\frac{\alpha_{i}\left(t-\tau_{i}\right)}{\sqrt{2\sigma_{i}^{2}}}\right)\right]$$

where *A* denote the peak amplitude, τ is the retention time, σ^2^ represent the signal variance, and α is the skew parameter. When distribution is unskewed, α = 0. Further examples are provided in Supporting Information, Section C.

This model was applied to the Δλ and Δ*I* traces for the PhCN-MRR, providing a systematic way to compare dynamic responses across concentrations and transduction magnitudes. The goal is not only to extract robust peak metrics, but also to quantify injection repeatability and the impact of flow dynamics on the sensor response envelope.

#### Cross-Signal Metric Analysis

Figure 6a,c shows representative skew-normal fits to the extracted Δλ and Δ*I* signals for 1 and 2 mg/mL glucose injections when transduced with the PhCN-MRR. The point markers (black, blue, red) denote the raw data, while the shaded regions show the fitted skew-normal curves.

**Figure 6:**
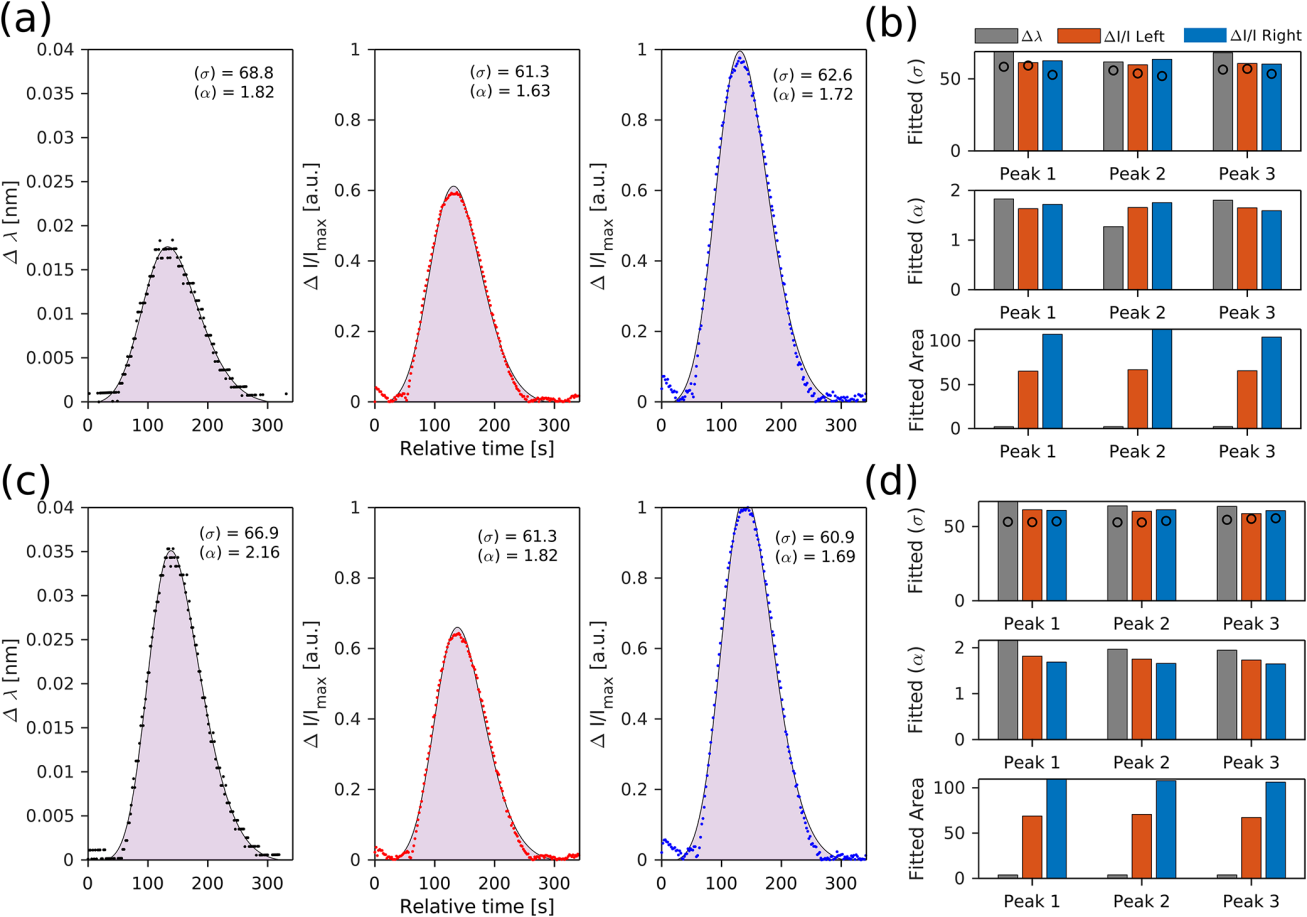
(a–c) FIA peaks corresponding to the 1 and 2 mg/mL glucose injections, respectively, for three transduction signals: wavelength shift (Δλ), and intensity changes at the left and right inflection points (Δ*I* left and Δ*I* right). The experimental data points are overlaid with fitted skew-normal function (shaded regions), capturing the full peak profile including asymmetry. (b–d) Extracted fitting parameters from (a, c), including peak area, width (σ), and skewness (α), shown for each transduction signal. Black circles overlaid on the width bars indicate the corresponding FWHM values obtained from the raw data for reference.

A summary of the fitted parameters across the three-transduction metrics: wavelength shift (Δλ), and intensity changes at the left and right inflection points (Δ*I* left and Δ*I* right), is shown in Figure 6b,d. Overlaid black circle markers indicate the full width at half-maximum (FWHM) as computed by the initial peak-finding algorithm, serving as a reference. In most cases, the fitted width (σ) agrees closely with the reference values across replicates, with deviations under 5%. Note that the fitted σ for Δλ is slightly higher than for the Δ*I*-based transduction, consistent across both concentrations in Figure 6a,c. This parameter, σ, characterizes the peak spread and is primarily influenced by dispersion during injection.

The skewness parameter (α) remained positive for all cases, confirming the expected right-tailed nature of the FIA peak, an indication of dispersion-dominated flow. Interestingly, α was consistently lower for intensity-based signals, reflecting a steeper rising edge and more gradual decay compared to the Δλ signal.

Across all replicates, the fitting process showed excellent agreement with experimental data, yielding average *R* ^2^ values exceeding 0.9. This confirms the skew-normal function as an effective model for capturing FIA transient dynamics under both Δλ and Δ*I* modalities.

Finally, the fitted area, a measure of signal strength, was the highest for the Δ*I* right signal. This trend aligns with the enhanced responsivity conferred by the Fano asymmetry in the PhCN-MRR, reinforcing the potential of asymmetric line shapes for maximizing transduction efficiency

### Dynamic Range Limitations

The dynamic measurements presented in this work demonstrate that both wavelength-based and intensity-based readouts from MRRs can reliably track analyte concentrations under continuous flow conditions. The PhCN-MRR, leveraging a Fano-shaped resonance, consistently outperformed the conventional MRR in terms of signal responsivity, particularly at the right inflection point (Δ*I* right), where asymmetry leads to steeper slopes. Further analysis could involve direct intensity measurements at the IP wavelengths to exploit this benefit more fully. Moreover, this enhanced performance highlights the advantage of asymmetric resonance line shapes for real-time detection, particularly considering that the resonance sampling rate (∼0.5 Hz) can be outpaced by tracking power variations at a fixed wavelength.

A key observation is the deviation in sensor response (Δ*I*) at higher analyte concentrations (e.g., 5–10 mg/mL), where the system begins to exceed the linear dynamic range of the resonance. As the induced refractive index change pushes beyond the regime where intensity and wavelength shift scale linearly, the Δ*I* response becomes distorted and no longer suitable for accurate quantification. Figure 7 provides an example for 5 mg/mL where this transition is clearly observable. Therefore, the price paid for increased responsivity is a reduced dynamic range, which, for the current setup, limits accurate Δ*I*-based detection to concentrations below ∼5 mg/mL. This contrasts with Δλ-based readout, which is primarily limited by the MRR’s free spectral range.

**Figure 7:**
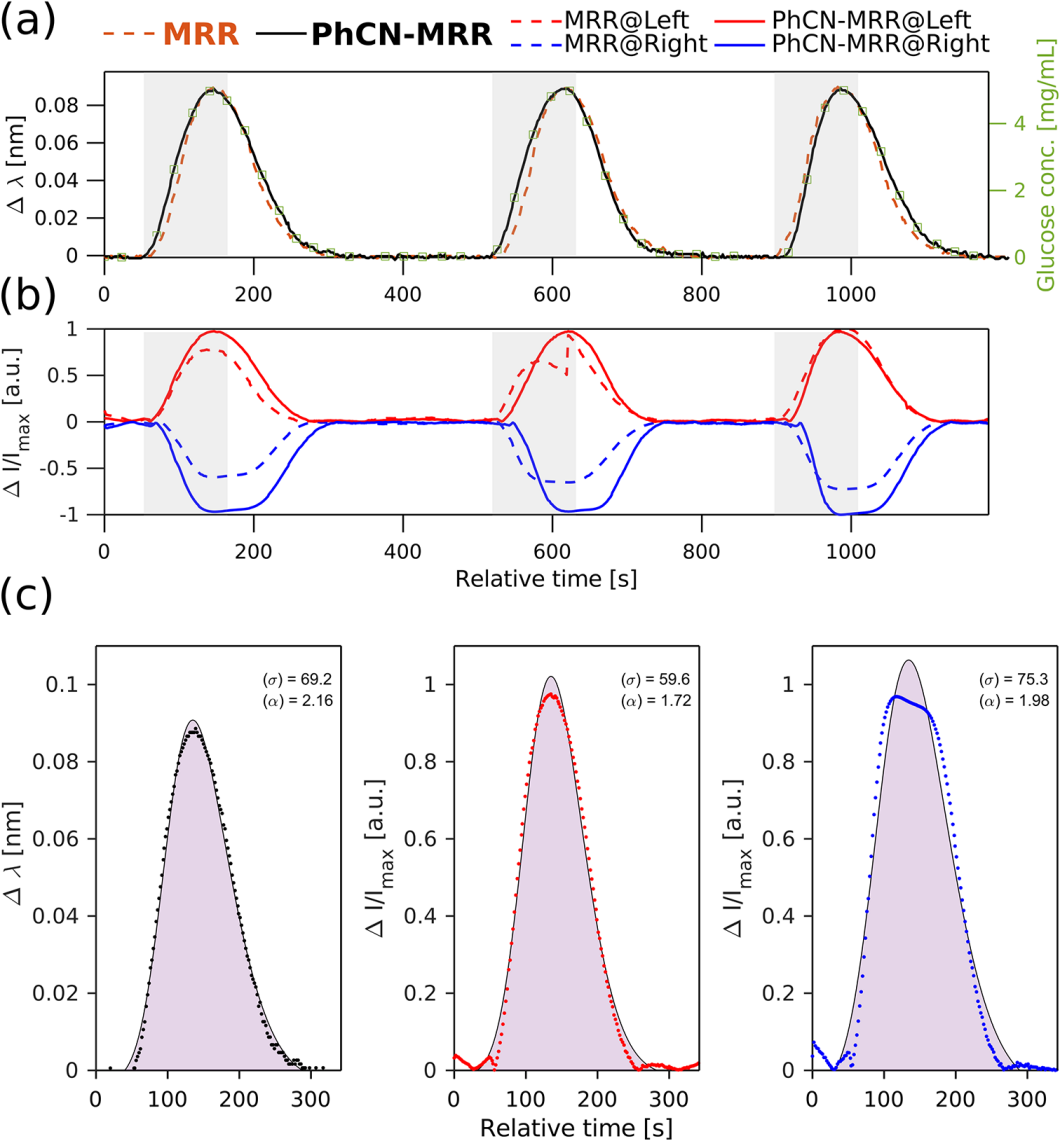
(a) Extracted wavelength shift (Δλ) over time for the PhCN-MRR and conventional MRR during representative 5 mg/mL glucose injections. The gray shaded region indicates the nominal injection window. The corresponding back-calculated concentration, derived from the Δλ calibration curve, is overlaid on the secondary *y*-axis. (b) Corresponding intensity changes (Δ*I*) over time from the same experiment shown in (a), evaluated at the left (red) and right (blue) inflection points of the resonance dip for each device. (c) FIA peaks for the 5 mg/mL glucose injection, plotted for all three transduction signals: Δλ, Δ*I* left, and Δ*I* right. Experimental data points are overlaid with skew-normal fits (shaded regions), illustrating peak width (σ) and asymmetry (α).

To further illustrate the limitations and characteristics of the PhCN-MRR under higher analyte concentrations, Figure 7 presents a detailed comparison of the transduction signals during representative 5 mg/mL glucose injections. In panel (a), the extracted wavelength shift (Δλ) over time shows a consistent and monotonic response across both the MRR and PhCN-MRR devices, with the back-calculated concentrations closely following the injection window. This confirms that Δλ remains within a linear and interpretable regime at this concentration. In contrast, panel (b) shows the corresponding intensity changes (Δ*I*) evaluated at the left and right inflection points of the Fano resonance. While Δ*I* right initially provides higher responsivity at lower concentrations, here it exhibits noticeable deviation (flatten peak) from expected linearity, including a distorted transient profile and possible saturation effects.

At concentrations above ∼5 mg/mL, the Δ*I* response is no longer linear, whereas Δλ remains strictly proportional to the RI change. This confirms that the deviation originates from the intensity-based readout itself: as the operating wavelength approaches the extreme of the asymmetric Fano resonance, the local slope progressively saturates, leading to compression of the Δ*I* signal at higher RI contrasts. The linear range of Δ*I* detection can be extended by adjusting the Fano asymmetry parameter *q*, modifying the slot or PhCN geometry to redistribute the slope, or operating at a slightly detuned wavelength lying on a more linear portion of the resonance. Nevertheless, Δ*I* remains the optimal transduction method for fast and low-contrast RI changes.

Figure 7c further supports this by comparing the fitted skew-normal curves for each transduction channel. While all signals can be fit using the model, the shape of the Δ*I* peaks, especially Δ*I* right. These distortions indicate that the sensor exceeds the linear dynamic range of the Fano-enhanced resonance, limiting the quantitative use of Δ*I* at this concentration. This analysis highlights the trade-off between increased responsivity and reduced dynamic range in intensity-based detection and reinforces the value of wavelength-tracking approaches (Δλ) for high-concentration sensing scenarios.

Unlike Δλ, Δ*I* detection is less directly constrained by the resonance linewidth, though it remains influenced by the slope and *Q* factor of the resonance. However, Δ*I* performance becomes increasingly limited by the noise floor and resolution of the optical power detection system. This shift emphasizes that, in high-responsivity sensing regimes, the limit of detection (LOD) becomes more dependent on system-level noise and power meter stability, rather than only the intrinsic optical properties of the resonator if, for instance, resonance broadening effect is negligible.

The skew-normal modeling of the FIA peaks adds a quantitative perspective to the interpretation of signal dynamics. The extracted peak σ and α parameters consistently captured injection dispersion and reflected the asymmetric flow profile induced by peristaltic pumping. Notably, Δ*I* signals exhibited slightly reduced skewness compared to Δλ, consistent with steeper rising edges and faster signal onset. This analysis provides a framework to benchmark the analytical content of the measured transients and supports future development based on signal morphology. Additionally, it quantifies transient behavior in a repeatable way, helping to define the temporal resolution of the sensor.

Repeatability across replicates and concentrations further validates the robustness of the PhCN-MRR platform. Our results demonstrate consistent transduction behavior over multiple injection cycles, going beyond isolated or static sensor outputs. This reproducibility brings the system closer to real-world analytical workflows, such as those encountered in high-performance liquid chromatography (HPLC). Importantly, the observed separation between the baseline noise floor and the transient signal suggests that intensity-based detection could outperform wavelength-tracking approaches at low concentrations, proved that optical power fluctuations are sufficiently minimized or compensated.

From a design standpoint, minimizing the LOD in MRR-based systems requires a careful balance between waveguide sensitivity and intrinsic quality factor (*Q*_*i*_). Wider waveguides reduce scattering losses and enhance *Q*_*i*_, while narrower ones increase evanescent field overlap, thereby improving refractive index sensitivity. In our design, a larger ring radius (e.g., *R*_MRR_ = 35 μm) was first employed to increase *Q*_*i*_, followed by the integration of a longer PhCN perturbation (e.g *N*_H_ = 10) to induce a Fano-shaped resonance and boost slope responsivity. Coupling gap engineering and reduction of sidewall roughness offer further opportunities to improve total quality factor (*Q*) and overall sensing performance.

Although the asymmetric resonance improves responsivity at one inflection point, a consistent reduction in intensity was observed on the opposite side. While this effect does not currently hinder sensing capabilities, it suggests an energy redistribution mechanism within the resonance profile that requires further investigation, particularly in the context of bidirectional sensing architectures.

To put the performance of the PhCN-MRR in context, Table 2 summarizes the reported metrics for experimentally demonstrated Fano-assisted microring resonator sensors operating in aqueous environments. This comparison is necessarily selective, as moving Fano resonances from fundamental demonstration to practical liquid-phase sensing remains a recognized challenge.^46^ As many Fano-based MRR demonstrations use air or oxide cladding and do not provide fluidic handling, only works reporting bulk aqueous RI measurements are included for comparison. Metrics are listed as reported by the respective authors.

**Table 2:** Experimental Fano-Assisted Sensing Architectures Based on MRRs for Aqueous RI-Sensing

device type	*q*-	*Q*-	sensitivity [nm/RIU]	LOD [mg/mL]	footprint^a^ [μm × μm]	flow rate [μL/min]	platform (thickness [μm])	ref
photonic crystal cavity-MRR	>0.5^c^	n/a	n/a^e^ ^,^ ^f^	0.24^f^	>∼31 × 31	stopped-flow	polymer (−)	^8^
subwavelength grating waveguide-MRR	>0.5^c^	1.3·10^4^	363^e^	n/a	∼11.5 × 11.5	stopped-flow	Si (0.220)	^47^
multimode waveguide-MRR	<0.5^c^	2.1–2.7·10^4^	1120/RIU^g^	n/a	∼310 × 304	stopped-flow	polymer (−)	^7^
**PhCN-MRR**	**0.2**	**1–1.5·10** ^**4**^	**111** ^e^	**0.0832** ^e^	**∼40 × 34**	**70** ^d^	**Si**_**3**_ **N**_**4**_ **(0.300)**	^b^
				**0.0238** ^f^				

n/a Not available / Not explicitly reported.

a Footprint, estimated area considering the radius, coupling gap and waveguide width.

b This work.

c Estimated Fano asymmetry value from spectral shape.

d Flow Injection (0.5–1 Hz).

e Δλ transduction.

f Δ*I* transduction.

g Δ*V* (visibility change) transduction.

## Conclusions

This study demonstrates the successful integration of a Fano-resonant Si_3_N_4_ photonic crystal nanobeam-assisted microring resonator (PhCN-MRR) into a microfluidic FIA platform for real-time refractive index sensing. Through sequential stopped-flow and dynamic injection tests across five glucose concentrations (0.5–10 mg/mL), the PhCN-MRR consistently delivered robust and repeatable responses in both Δλ and Δ*I* modalities showing a spectral sensitivity of ∼111 nm/RIU like the ∼113 nm/RIU from a MRR. This confirms that the hybrid photonic-crystal coupling preserves bulk RI sensitivity while introducing a controllable resonance asymmetry.

Importantly, intensity-based detection provided enhanced responsivity at low concentrations (<5 mg/mL), achieving a lower limit of detection (LOD) and quantification (LOQ). This improvement was balanced by reduced linearity at higher concentration, where Δλ maintained accuracy remaining linear up to 10 mg/mL. To interpret dynamic flow responses, we applied skew-normal modeling to the FIA peaks. Extracted parameters such as peak width (σ) and skewness (α) reliably captured the effects of injection dispersion and flow asymmetry. This quantitative framework enables direct comparison of transduction mechanisms (Δλ, Δ*I*) and could support algorithmic peak deconvolution or concentration estimation in future analytical sensing workflows.

The system also exhibited excellent repeatability across replicates and concentrations, confirming its potential for reliable dynamic sensing, an aspect often underreported in photonic sensor studies. These results position the PhCN-MRR as a promising platform for label-free, time-resolved biochemical or environmental monitoring applications, where both sensitivity and signal fidelity are critical. Although the PhCN-MRR studied here was not spectrally optimized for maximum Fano asymmetry, its CMOS-compatible fabrication, compact footprint, and demonstrated dual-mode transduction establish a strong foundation for lab-on-a-chip integration.

Furthermore, the demonstrated performance suggests applicability in microfluidic chemical analysis, small-volume environmental monitoring, and point-of-care diagnostics, in which bulk-RI signatures can provide useful insights. The intrinsic compatibility of Si_3_N_4_ resonators with photothermal spectroscopy, where absorption-induced thermo-optic shifts generate molecularly specific contrasts, further expands the relevance of the PhCN-MRR toward integrated photothermal detection in the visible or mid-IR domain.^48–50^ Taken together, these attributes establish the PhCN-MRR architecture as a versatile building block for next-generation on-chip analytical technologies.

Future efforts will focus on device and application-level extension of this approach. At the device level, we will pursue optimized Fano designs (optimal *Q*, *q*, ER) and explore reflection-mode operation for broader applicability within lab-on-a-chip and optical metrology systems. At the application level, we aim to systematically compare Δλ and Δ*I* based transduction across a broader range of analytes, including salts, proteins and small molecules, to assess performance in increasingly complex biological and chemical matrices.

## Supplementary Material



## Data Availability

Data underlying the results presented in this paper are not publicly available at this time but may be obtained from the authors upon reasonable request.

## Notes

Refractometric sensing setup, wavelength shift and intensity shift dynamics, Skew-Gaussian fitting, Resonant mode filed distribution (PDF)

## Abbreviations

CMOS: complementary metal-oxide-semiconductor
FIA: flow injection analysis
PhCN: photonic crystal nanobeam
RI: refractive index
SD: standard deviation of residuals
CV: coefficient of variation
LOD: limit of detection
LOQ: limit of quantification
MRR: microring resonator
