# Supplementary material for: Flow Injection-Based Refractive Index Sensing with a Si3N4 Photonic Crystal Nanobeam-Microring Fano Resonator
Source: ACS Appl Opt Mater. 2025 Dec 9;3(12):2946–59. doi: 10.1021/acsaom.5c00500 (PMC12751107; doi:10.1021/acsaom.5c00500)
Supplement: Supplementary file 1 [file ot5c00500_si_001.pdf]

Flow Injection-Based Refractive Index Sensing with a Si_3N_4 Photonic Crystal Nanobeam-Microring Fano Resonator: Supporting Information.

JESUS HERNAN MENDOZA-CASTRO [1,2,*], SILVIA SCHOBESBERGER[3], ARTEM S. VOROBEV [1,4,5], SIMONE IADANZA[4,6,7], GIOVANNI MAGNO[1], LIAM O'FAOLAIN [4,5], BERNHARD LENDL[2,*] AND MARCO GRANDE [1]

[1]*Department of Electrical and Information Engineering, Politecnico di Bari, Via E. Orabona, 4, 70126 Bari, Italy*
[2]*Institute of Chemical Technologies and Analytics, TU Wien, Getreidemarkt 9/164, Vienna, 1060, Austria*
[3]*Institute of Applied Synthetic Chemistry,TU Wien, Getreidemarkt 9/163, Vienna, 1060, Austria*
[4]*Centre for Advanced Photonics and Process Analysis, Munster Technological University, T12 T66T Bishopstown, Cork, Ireland*
[5]*Tyndall National Institute, T12 PX46 Cork, Ireland*
[6]*Laboratory of Nano and Quantum Technologies, Paul Scherrer Institut, 5323 Villigen, Switzerland*
[7]*Laboratory of Integrated Nanoscale Photonics and Optoelectronics, École Polytechnique Fédérale de Lausanne, 1015 Lausanne, Switzerland*
* *jesus.mendoza-castro@tuwien.ac.at ; bernhard.lendl@tuwien.ac.at*

Abstract: This manuscript provides additional supporting information to complement the content presented in the main manuscript. It includes further detailed experimental results presented offering a deeper understanding of the methodologies and analyses performed.

Keywords: Fano resonances, Flow Injection Analysis, refractive index sensing, microfluidics, silicon nitride.

A. Refractometric sensing setup

Figure. S1 provides a detailed overview of the experimental setup used for the characterization of the Si_3N_4-based MRR and PhCN-MRR devices under both static and dynamic conditions.

1.1 Optical Readout Configuration

A semiconductor tunable laser (TSL) paired with an InGaAs photodetector for high-resolution transmission scans. Light polarization was controlled via a fiber polarization controller (FPC) to excite the TE-like waveguide mode. The collimated beam was focused onto the input facet using a pair of microscope objectives (60×), while the output light was collected and directed through free-space optics or fibers into the detection system. An apochromatic microscope objective (AO) is used for inspection of the scatter light in the chip and precise alignment. The entire system was thermally stabilized using a Peltier-cooled stage set to 20 °C to minimize temperature-induced resonance drift.

1.2 Optical Readout Configuration

A custom microfluidic flow-injection setup was designed to deliver time-resolved analyte injections over the photonic transducer. The system incorporated a 6-port injection valve, a 100 µL sample loop, and a peristaltic pump operating at 70 µL/min. Manual switching of the valve allowed sequential sample introduction. A minimum interval of 4 minutes was enforced between injections to allow for full exchange of sample volumes.

During continuous flow, RI fluctuations could arise from dispersion during injection and incomplete plug formation at low concentrations. A laminar, low-pulsation peristaltic pump and a fixed injection volume were used to minimize injection-to-injection variability.

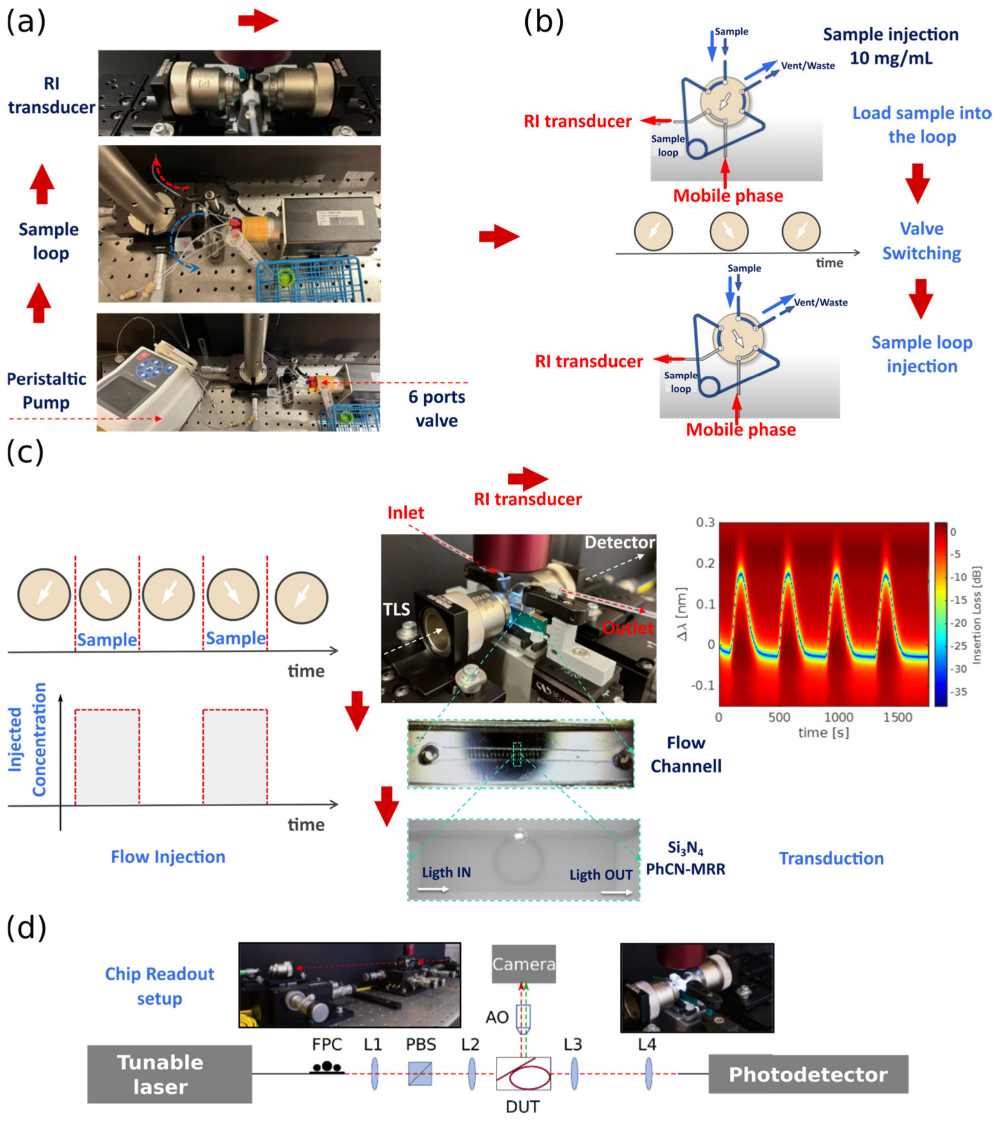

Figure. S1 (a) Liquid handling setup used for flow injection analysis (FIA). (b) Operation of the 6-port valve for generating fixed-volume analyte injections. (c) Workflow diagram summarizing the FIA experiment cycle, including analyte loading, injection, chip inspection and spectral acquisition. (d) Optical readout setup for characterizing the Si_3N_4 photonic chips. L_1 and L_4 are 10× collimation lenses; L_2 and L_3 are 60× focusing lenses. AO denotes the apochromatic microscope objective, integrated with tube lenses and connected to a short-wave infrared (SWIR) camera. FPC: fiber polarization controller. PBS: polarization beam splitter. DUT: device under test. Green dashed lines represent free-space optical paths for visible wavelengths, while red dashed lines indicate paths for near-infrared wavelengths. Solid and dotted lines show the guided light paths through fiber optics.

Each analyte concentration was tested in 5 injections to assess repeatability. Blank injections with deionized water (DIW) were performed as controls to verify system stability and baseline drift.

1.3 Spectral Acquisition and Data Timing

The TSL scanned over the resonance at 100 nm/s, enabling full transmission spectra to be acquired every 1-2 seconds. This acquisition rate was sufficient to resolve dynamic signal variations during flow injection cycles. The injection window was designed to show signal transients on a ~60-100 s timescale, depending on dispersion and analyte properties, corresponding to the chosen flow rate of 70μL/min.

1.4 Cleaning and Preparation Protocols

The filled channel was confirmed to cover the resonator region using the integrated SWIR imaging system. After each measurement, the chip was thoroughly rinsed with DIW to maintain surface cleanliness and reproducibility. The same cleaning protocol was conducted between flow-injection experiments.

B. Wavelength shift and Intensity shift Dynamics

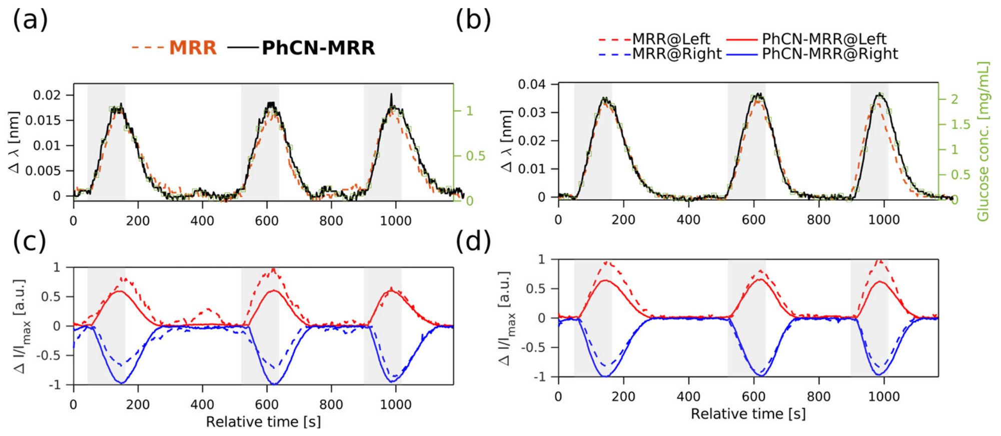

Figure S2. (a-b) Extracted wavelength shifts ($\Delta\lambda$) over time for the PhCN-MRR and MRR devices during representative 1 mg/mL and 2 mg/mL glucose injections. Gray shaded regions indicate the nominal injection window, while the back-calculated concentration from the $\Delta\lambda$ calibration is overlaid on the secondary *y*-axis. (c-d) Corresponding intensity changes (ΔI) over time from the same datasets as in (a–b), evaluated at the left (red) and right (blue) inflection points of the resonance dip for each device. The shaded regions again represent the expected injection period.

To evaluate the dynamic response of the devices, we analyzed the temporal evolution of the $\Delta\lambda$ and ΔI under controlled glucose injections. As shown in Figure S2(a-b), the extracted $\Delta\lambda$ traces for 1 mg/mL and 2 mg/mL glucose injections reveal a clear concentration-dependent response for both the conventional MRR (dashed lines) and the PhCN-MRR (solid lines). The complementary y-axis (green right) presents the back-calculated concentration derived from the $\Delta\lambda$ calibration curve. As expected, the glucose concentration roughly double $\Delta\lambda$, indicating linear sensitivity within the given range. At both concentrations, the PhCN-MRR consistently exhibits a slightly higher response than the conventional MRR, suggesting enhanced sensitivity. Overall, both sets of results shown in Figure S2(a-b) align with the sensitivity trends observed in Fig. 4(c) in the main manuscript. Each injection was repeated at least three times to ensure measurement repeatability. At lower concentrations, however, the resolution step of the tunable laser source (1 pm in this setup) becomes a limiting factor (Fig. 4(c) main manuscript).

Figure S2(c-d) presents complementary analysis of the intensity variation at the resonance inflection points. The MRR (dashed lines) displays nearly symmetric modulation at the left (red) and right (blue) sides of the resonance, while the PhCN-MRR (solid lines) shows a more pronounced asymmetric intensity response, consistent with the expected Fano lineshape. At higher concentrations (e.g., 10 mg/mL, not analyzed in detail here), the resonance response

enters a non-linear regime, where the wavelength shift approaches to the pseudopeak edges resonance, leading to distorted ΔI signals and reduced transduction fidelity.

Baseline fluctuations are more pronounced for the ΔI case than for $\Delta\lambda$. The dominant source of baseline variation appears to be thermal drift ($\Delta\lambda$), alignment drift and Fabry-Perot fringes introduced by residual reflections in the fiber-chip coupling (ΔI). Flow-induced disturbances could contribute to additional low-frequency drift, not visible in the current setup. Despite these factors, the baseline remained stable across replicates, indicating good reliability for dynamic sensing.

These findings highlight the promise of intensity-based transduction, particularly in Fano-resonant systems, as a sensitive method for low-concentration detection when combined with time-resolved analysis.

C. Skew-Gaussian Fitting

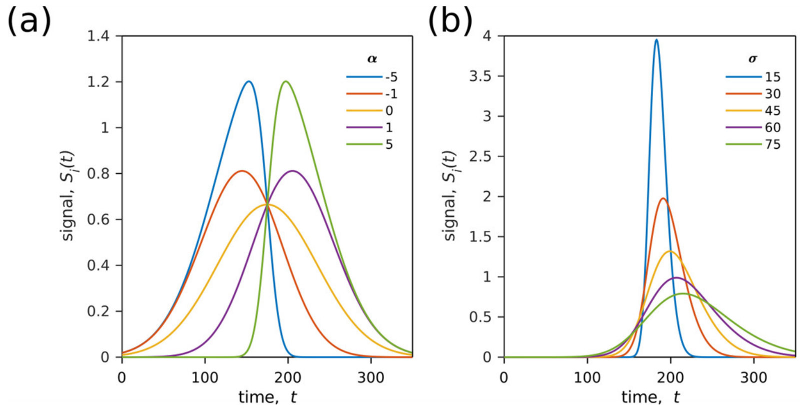

Figure S3. Parametric illustration of the skew-normal model behavior under controlled parameter variations. (a) Simulated peak profiles generated by varying the skewness parameter (α) while keeping the width fixed at $\sigma = 60$. Increased $\alpha > 0$ results in increasingly right-tailed peaks, mimicking dispersion-dominated FIA profiles. (b) Simulated peaks showing the effect of varying σ (peak width) with a fixed skewness $\alpha = 2$. Broader σ values lead to flatter, more dispersed peak shapes. These plots illustrate how the skew-normal model captures key characteristics of flow-induced peak distortions, justifying its use in fitting experimental FIA signals.

To better understand how the skew-normal model captures the shape of flow-injection peaks, we visualize the influence of individual parameters based on Equation (2) from the main manuscript. Figure S3 presents a parametric sweep of the skewness (α) and width (σ) parameters, while keeping the other variables constant.

The total time window is set to 350 seconds, matching the reference frame used in the experimental plots shown in Fig. 6 of the main manuscript. The amplitude (A), peak center (τ), and vertical offset are fixed at 100, 175 seconds, and 0, respectively. Under these fixed conditions, we systematically vary: Skewness α (Figure S3(a)), while keeping $\sigma = 60$; Width σ, while fixing $\alpha = 2$ (Figure S3(b)).

These parametric insights demonstrate how changes in α and σ modulate peak asymmetry and broadening, respectively. This analysis supports the application of the skew-normal function for fitting FIA signals, as it effectively captures the key morphological features observed in experimental data. Moreover, Figure S3 supports the interpretability of fitted values extracted from experimental data. It presents the potential of skew-normal modeling for algorithmic peak deconvolution or concentration estimation based on transient morphology, especially in the discussed ($\Delta\lambda/\Delta I$) transduction systems for application in High Performance Liquid Chromatography (HPLC)[1].

D. Resonant Mode Field Distribution

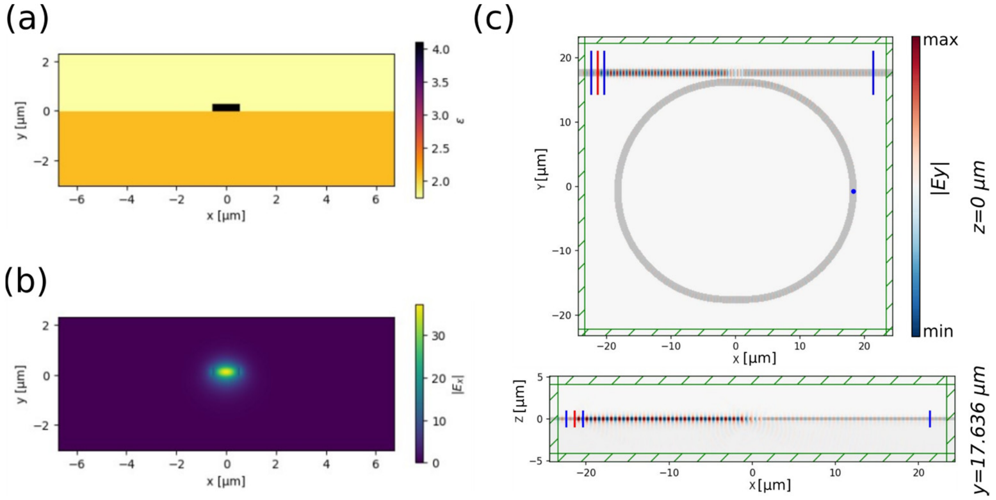

Figure S4. (a) Relative permittivity distribution of the Si_3N_4 MRR waveguide cross-section. (b) Simulated guided-mode intensity profile of the fundamental TE-like mode. (c) Simulated spatial distribution of the electric-field component E_y in the PhCN-MRR, shown from the top view and side view. These simulations illustrate the confinement of the guided mode within the Si_3N_4 core, and the local field modulation introduced by the PhCN section, which enables the Fano-shape interference observed experimentally. The green hatched boxes surrounding the simulation domain correspond to the perfectly matched layers (PMLs). The red line marks the location of the excitation source, and the blue lines indicate the positions of the flux monitors.

The fundamental TE-like guided mode field distribution is computed by the plane wave expansion method through open-source software package: MPB[2]. The relative permittivity distribution of the waveguide cross section immersed in DIW is shown in Figure S4(a). The resulting electric-field intensity shown in Figure S4(b) corresponds to the lowest-order TE-like eigenmode and is used to illustrate the modal confinement within the waveguide core.

The spatial field distribution in the PhCN-MRR device was computed with the finite-difference time-domain (FDTD) method[3], using an open-source software package: MEEP[4]. The full 3D geometry of the structure, including the bus waveguide, PhCN section, and MRR, was modelled with the same material indices used in the eigenmode calculations. A Gaussian-modulated eigenmode source placed in the bus waveguide, spectrally centered around the resonance wavelength of interest (e.g. 1564 nm) with a full width at half maximum of 2 nm.

Although this broadband excitation does not represent the steady-state CW cavity field, it enables efficient excitation of the resonance and provides qualitatively accurate spatial localization patterns. The top- and side-view intensity maps shown in Figure S4(c) correspond to the $|E_y|$ distribution at the resonance wavelength and illustrate the localization of the optical field within the PhCN coupling region and the MRR.

References

(1) Mendoza-Castro, J. H.; Tomasetig, D.; Schobesberger, S.; Vorobev, A. S.; Ricchiuti, G.; Dabrowska, A.; Iadanza, S.; Grande, M.; Magno, G.; O'Faolain, L.; Lendl, B. Photonic Integrated Circuits for Refractive Index-Based Detection in Liquid Chromatography. In *2025 IEEE Silicon Photonics Conference (SiPhotonics)*; 2025; pp 1–2. https://doi.org/10.1109/SiPhotonics64386.2025.10985307.

(2) Johnson, S. G.; Joannopoulos, J. D. Block-Iterative Frequency-Domain Methods for Maxwell's Equations in a Planewave Basis. *Opt. Express* **2001**, *8* (3), 173–190. https://doi.org/10.1364/OE.8.000173.

(3) Taflove, A.; Oskooi, A.; Johnson, S. G. *Advances in FDTD Computational Electrodynamics: Photonics and Nanotechnology*; Artech House, 2013.

(4) Oskooi, A. F.; Roundy, D.; Ibanescu, M.; Bermel, P.; Joannopoulos, J. D.; Johnson, S. G. Meep: A Flexible Free-Software Package for Electromagnetic Simulations by the FDTD Method. *Comput. Phys. Commun.* **2010**, *181* (3), 687–702. https://doi.org/10.1016/j.cpc.2009.11.008.
